# Elevated HOXA1 expression correlates with accelerated tumor cell proliferation and poor prognosis in gastric cancer partly via cyclin D1

**DOI:** 10.1186/s13046-016-0294-2

**Published:** 2016-01-21

**Authors:** Chenwei Yuan, Xingwu Zhu, Yang Han, Chenlong Song, Chenchen Liu, Su Lu, Meng Zhang, Fudong Yu, Zhihai Peng, Chongzhi Zhou

**Affiliations:** Department of General Surgery, Shanghai General Hospital, Shanghai Jiaotong University School of Medicine, Shanghai, 200080 P. R. China; Department of Pathology, Shanghai General Hospital, Shanghai Jiaotong University School of Medicine, Shanghai, 200080 P. R. China; Department of General Surgery, Kashgar Prefecture Second People’s Hospital, Kashgar, Xinjiang Uyghur Autonomous Region 844000 P. R. China

**Keywords:** HOXA1, cyclin D1, Gastric cancer, Biomarker, Prognosis

## Abstract

**Background:**

HOXA1 is a member of the Homeobox gene family, which encodes a group of highly conserved transcription factors that are important in embryonic development. However, it has been reported that HOXA1 exhibits oncogenic properties in many malignancies. This study focused on the expression and clinical significance of HOXA1 in gastric cancer (GC).

**Methods:**

To assess the mRNA and protein expression of HOXA1 and cyclin D1 in GC tissues, we utilized qRT-PCR and western blotting, respectively. The effects of HOXA1 on GC cell proliferation, migration, and invasion, as well as xenograft tumor formation and the cell cycle were investigated in our established stable HOXA1 knockdown GC cell lines. The protein expression of HOXA1 and cyclin D1 was examined by immunohistochemistry using GC tissue microarrays (TMA) to analyze their relationship on a histological level. The Kaplan-Meier method and cox proportional hazards model were used to analyze the relationship of HOXA1 and cyclin D1 expression with GC clinical outcomes.

**Results:**

HOXA1 mRNA and protein expression were upregulated in GC tissues. Knockdown of HOXA1 in GC cells not only inhibited cell proliferation, migration, and invasion in vitro but also suppressed xenograft tumor formation in vivo. Moreover, HOXA1 knockdown induced changes in the cell cycle, and HOXA1 knockdown cells were arrested at the G1 phase, the number of cells in S phase was reduced, and the expression of cyclin D1 was decreased. In GC tissues, high cyclin D1 mRNA and protein expression were detected, and a significant correlation was found between the expression of HOXA1 and cyclin D1. Survival analysis indicated that HOXA1 and cyclin D1 expression were significantly associated with disease-free survival (DFS) and overall survival (OS). Interestingly, patients with tumors that were positive for HOXA1 and cyclin D1 expression showed worse prognosis. Multivariate analysis confirmed that the combination of HOXA1 and cyclin D1 was an independent prognostic indicator for OS and DFS.

**Conclusion:**

Our data show that HOXA1 plays a crucial role in GC development and clinical prognosis. HOXA1, alone or combination with cyclin D1, may serve as a novel prognostic biomarker for GC.

## Background

Gastric cancer (GC) is a leading cause of cancer-related death worldwide [1, 2], and it is also the third most common cancer and a major cause of cancer-related death in China [3]. Although much progress has been made in the diagnosis and treatment of GC in recent years, the survival rate remains unsatisfactory (20–25 %) [4], which may be attributable in part to late diagnosis and the postoperative recurrence or metastasis of primary GC. There are no distinct symptoms of GC, and the current tumor markers are of little use for prognostic evaluation [5, 6]. Thus, the identification of new useful biomarkers for early detection and prognosis in patients with GC is of great importance.

HOX genes, which were first discovered in *Drosophila melanogaster* mutants in the early 1900s, constitute a highly conserved subgroup of the homeobox superfamily that encodes transcription factors with a 60-amino acid domain called the homeodomain. HOX genes play important roles in embryonic development by regulating numerous processes, including cell proliferation, apoptosis, differentiation, angiogenesis, and so on [7–9]. In mammals, there are 39 HOX genes, which are located in 4 chromosomal clusters, referred to as *HOXA*, *B*, *C*, and *D*, that each contain 9–11 genes [9]. During normal vertebrate development, HOX genes play an important role in the mechanisms underlying the following three basic precepts: spatial collinearity, posterior prevalence, and temporal collinearity [8]. However, aberrant expression of HOX genes occurs in many cancers, such as acute leukemia, lung cancer, and cervical carcinoma, among others [10–14].

In our previous studies, we detected several differentially expressed HOX genes in GC using high-throughput cDNA microarrays [15]. Among these genes, *HOXA1* was more highly expressed in 8 out of 12 tumor tissues than in normal tissues[15]. *HOXA1* is a part of the A cluster on chromosome 7, and it encodes a DNA binding transcription factor that regulates the expression of genes involved in morphogenesis, cell proliferation, and differentiation [16–18]. Some studies have demonstrated the roles that HOXA1 plays in tumorigenesis. Brock et al. [19] showed that HOXA1 is a critical mediator of mammary tumor progression in humans. A recent study showed that loss of HOXA1 impairs cellular progression by blocking the G1-S transition in HeLa cells [20]. In addition, Zhang et al. [21] demonstrated that ectopic expression of HOXA1 in MCF7 breast cancer cells upregulates cyclin D1. Interestingly, cyclin D1 has been found to be highly expressed in GC [22, 23] and many other malignancies such as breast cancer [24] and cutaneous melanoma [25]. Cyclin D1 is well known for its role in the response to the mitogenic signals that promote progression through the G1-S checkpoint of the cell cycle [26]. Recently Seo et al. [27] reported that downregulation of cyclin D1 in GC cells by a lentivirus significantly inhibited cell function and motility in vitro, and significantly inhibited cancer growth when engrafted into nude mice. However, the relationship between HOXA1 and cyclin D1 in GC has not been elucidated in detail.

The current study aimed to investigate the expression and clinical significance of HOXA1 in GC. First, we assessed the expression of HOXA1 in GC at both the transcriptional and translational levels. Second, we studied the effects of HOXA1 on GC cell proliferation, migration, invasion, cell cycle progression, and xenograft tumor formation by knocking down the expression of HOXA1, and we found that the expression of cyclin D1 was also decreased. Third, we determined the mRNA and protein expression of cyclin D1 in GC to examine the relationship between HOXA1 and cyclin D1. Finally, we investigated the relationship of HOXA1 and cyclin D1 with clinical characteristics and the prognostic value of HOXA1, either alone or in combination with cyclin D1, using GC tissue microarrays (TMA). We found that HOXA1 plays a role in the development and clinical prognosis of GC, and it may be useful as a novel prognostic biomarker for GC, either alone or in combination with cyclin D1.

## Methods

### Patients and specimens

Fresh primary cancer and paired adjacent normal tissue specimens were collected from 48 GC patients (33 males and 15 females) in the Department of General Surgery of Shanghai General Hospital. The tissues were collected after surgical resection, frozen immediately in liquid nitrogen, and stored at −80 °C until RNA and protein extraction. A total of 264 preserved human GC tissue specimens (from 157 males and 107 females) from Shanghai General Hospital were paraffin embedded for TMA construction. Disease-free survival (DFS) and overall survival (OS) were defined as the interval from surgery to clinically or radiologically proven recurrence/metastasis and death, respectively. Tumor staging was based on pathological outcomes according to the guidelines of the International Union against Cancer (UICC) [28], and the diagnoses were confirmed by two pathologists. Patients who had never received chemotherapy or radiotherapy were enrolled in this study. All of the patients provided written informed consent. The study was approved by the Ethics Committee of Shanghai General Hospital.

### Cell lines and the establishment of HOXA1 knockdown cell lines

The human gastric cancer cell lines HGC-27, SGC-7901, MGC-803, BGC-823, and AGS were purchased from the Type Culture Collection of the Chinese Academy of Science (Shanghai, China). Cells were cultured in 1640/F12k medium supplemented with 10 % fetal bovine serum (FBS; Gibco, Australia) and 1 % penicillin–streptomycin at 37 °C in a humidified atmosphere containing 5 % CO_2_.

The HOXA1 RNAi lentiviral expression plasmid was purchased from Scigebio Biotechnology Co., Ltd. (Shanghai, China). The oligonucleotides used to generate the shRNA targeting HOXA1 (shHOXA1) were forward: AGTTATCTTAGCTGGATATAA and reverse: TTATATCCAGCTAAGATAACT. SGC-7901 and BGC-823 cells were transfected with 1 × 10^8^ transducing units/mL of lentivirus particles. The cells were subjected to antibiotic selection (with 1.2 μg/mL puromycin) after transfection. As a result, two stable cell lines (SGC-7901-shHOXA1 and BGC-823-shHOXA1) were established. Cells transfected with a control shRNA sequence were used as controls (SGC-7901-Control and BGC-823-Control).

### RNA extraction and quantitative real-time PCR (qRT-PCR)

Total RNA from GC cells and tissue specimens was extracted with TRIzol reagent (Invitrogen, NY, USA) according to the manufacturer’s instructions, and 2 μg of total RNA from each sample were reverse transcribed into cDNA using PrimeScript™ RT Master Mix (Perfect Real Time; Takara, Shiga, Japan) according to the manufacturer’s instructions. qRT-PCR was performed using SYBR® Premix Ex Taq™ (Tli RNaseH Plus; Takara) in a ViiA™ 7 Real-time PCR System (Applied Biosystems, NY, USA) according to the manufacturer’s protocol. The amplification was performed as follows: an initial denaturation step for 2 min at 95 °C followed by 40 cycles of denaturation for 10 s at 95 °C, annealing for 30 s at 59 °C, and elongation for 30 s at 72 °C, and a final extension step at 72 °C for 30 s. The specific primers used were as follows: HOXA1 sense, 5'-CGGCTTCCTGTGCTAAGTCT-3' and antisense, 5'-TTCATTGTGCCATCCATCAC-3'; cyclin D1 sense, 5'-GTGTATCGAGAGGCCAAAGG-3' and antisense, 5'-GCAACCAGAAATGCACAGAC-3'; and β-actin sense, 5'-CATGTACGTTGCTATCCAGGC-3' and antisense, 5'-CTCCTTAATGTCACGCACGAT-3'. β-Actin was used as an internal control, and the relative quantities (Δ cycle threshold [Ct] values) of each transcript were normalized to β-actin. Each reaction was repeated in triplicate. The fold changes (2^−ΔΔCt^) in *HOXA1* and *cyclin D1* mRNA expression were calculated using the following formulae: HOXA1ΔCt = (Avg. HOXA1_Ct - Avg. β-actin_Ct), HOXA1ΔΔCt = (HOXA1ΔCt_tumor - HOXA1ΔCt_non-tumor); cyclin D1ΔCt = (Avg. cyclin D1_Ct - Avg. β-actin_Ct), cyclin D1ΔΔCt = (cyclin D1ΔCt_tumor - cyclin D1ΔCt_non-tumor).

### Protein extraction and western blotting

Total protein was isolated from GC cells and tissue specimens using RIPA Lysis Buffer (Beyotime Biotechnology, Jiangsu, China). Protein concentration was measured using the BCA protein assay kit (Beyotime Biotechnology). Samples containing 40 μg of protein were separated by 10 % sodium dodecyl sulfate-polyacrylamide gel electrophoresis (SDS-PAGE), and the separated proteins were then transferred onto PVDF membranes. The membranes were blocked in 5 % fat-free milk solution containing 0.1 % Tween-20 for 1 h at room temperature. Membranes were then incubated with an anti-HOXA1 antibody (1:1,000; Abcam, Cambridge, UK), an anti-cyclin D1 antibody (1:100; Abcam), and an anti-α/β-tubulin antibody (1:1,000; Cell Signaling Technology, MA, USA) at 4 °C overnight. Next, the membranes were incubated with a secondary antibody (1:10,000; Jackson ImmunoReasearch Inc., PA, USA) conjugated to horseradish peroxidase for 1 h at room temperature. After washing with TBST buffer, the bands were visualized with Immobilon™ Western Chemiluminescent HRP Substrate (Millipore, MA, USA) according to the manufacturer’s instructions.

### Cell counting Kit-8 (CCK-8) assay

To observe the effect of HOXA1 knockdown on cell proliferation in vitro, the CCK-8 assay (Dojindo, Kumamoto, Japan) was employed to generate cell growth curves. Briefly, cells were plated in triplicate in 96-well cell culture plates at a density of 2,000 cells/well. At various time points (24, 48, 72, 96, and 120 h), the cells were incubated with 10 μL of CCK-8 solution for 2 h at 37 °C, and then the absorbance at 450 nm was measured on a Gen5 microplate reader (BioTek, VT, USA). The experiment was performed independently in triplicate.

### Plate colony formation assay

To evaluate colony formation, 800 log-phase cells were seeded in 6-well plates. After a 14-day incubation, the cells were fixed in methyl alcohol for 15 min and dyed with crystal violet for 15 min. Colonies were then counted, and the plates were photographed. The experiment was performed independently in triplicate.

### Migration and invasion assays

For these assays, 1 × 10^5^ cells in serum-free medium were seeded in the upper compartment of a transwell chamber (Millipore). The transwell membrane was either coated with Matrigel (for invasion; BD, CA, USA) or without (for migration). Then, the lower chamber was filled with 600 μL of basal medium containing 10 % FBS. After incubation at 37 °C in a humidified incubator containing 5 % CO_2_ for 24 h, the migrated or invaded cells on the lower membrane were fixed with methanol, stained with crystal violet, and then counted. The experiment was performed independently in triplicate.

### Nude mice xenograft models

Four-week-old male BALB/C nude mice were used to establish GC xenografts. The mice were then randomly divided into 2 groups (*n* = 5), and 5 × 10^6^ BGC-823-shHOXA1 or BGC-823-Control cells suspended in 100 μL of PBS were subcutaneously injected into their flanks. All mice were sacrificed 21 days after injection. Then, tumor mass and size were measured. Tumor volume was calculated using the following formula: volume = width^2^ × length × 0.5 [29]. Paraffin sections of the xenograft tissues were prepared for hematoxylin and eosin (H&E) staining and IHC with an anti-Ki67 antibody (1:500; Abcam). All animal studies were performed in accordance with the Shanghai General Hospital Animal Care guidelines. All efforts were made to minimize animal suffering.

### Flow cytometry analysis of the cell cycle

The Cell Cycle Kit (BD) was utilized to analyze the cell cycle. Cells were harvested, washed in phosphate buffered saline (PBS) for 5 min, and collected by centrifugation at 1,000 rpm for 10 min. Then, 5 mL of prechilled 70 % ethanol was used to fix the cells overnight at 4 °C. The cells were then washed, centrifuged, and resuspended in 0.5 mL of PI/RNase Staining Buffer. After incubation for 15 min, the cells were analyzed by flow cytometry (BD Accuri). The experiment was performed independently in triplicate.

### 5-Ethynyl-2′- deoxyuridine (EdU) incorporation assay

The EdU incorporation assay was performed using the Cell-Light™ EdU Apollo® 643 In Vitro Imaging Kit (RiboBio, Guangzhou, China) according to the manufacturer’s instructions. Cells were seeded on confocal dishes; when they reached about 50 % confluency, EdU labeling medium was added, and the cells were incubated for about 2 h. The cells were then fixed with 4 % formaldehyde for 30 min and treated with 0.5 % Triton X-100 for 30 min at room temperature. After washing three times with PBS, the cells were dyed with Apollo for 30 min. Then, Hoechst 33342 (5 μg/mL) was used to dye the DNA in the cells, and the cells were visualized with a confocal microscope. The experiment was performed independently in triplicate.

### Tissue microarray (TMA) construction and immunohistochemistry (IHC)

The method used to construct the TMA was previously reported [15]. After dewaxing and rehydration, the paraffin-embedded sections were drowned in boiled citrate buffer (0.01 M, pH 6.0) for antigen retrieval. Next, an anti-HOXA1 antibody (1:100; Abcam) and anti-cyclin D1 antibody (1:50; Abcam) were incubated with the slides at 4 °C overnight. Then, the anti-mouse or anti-rabbit EnVision™ two-step Visualization System (Gene Tech, Shanghai, China) was used to detect the primary antibody for 30 min at room temperature. Finally, the slides were counterstained with Mayer’s hematoxylin and covered with coverslips.

### Evaluation of immunostaining

We semi-quantitatively scored the expression levels of HOXA1 and cyclin D1 by calculating the staining intensity and area, according to the method of Han et al. [15], with slight modifications. The staining intensity was scored as 0 (negative), 1 (weak), 2 (moderate), or 3 (strong). The staining area was scored as 0 (0 %), 1 (1–25 %), 2 (26–50 %), or 3 (51–100 %) based on the percentage of positively stained cells. The final immunostaining score (IS) for each case was calculated by adding the staining intensity score to the staining area score. According to the IS, our specimens were divided into 2 groups as follows: positive HOXA1 and cyclin D1 expression was defined as an IS ≥3 (3, 4, 5, and 6), and negative expression was defined as an IS <3 (0 and 2).

### Statistical analysis

All statistical analyses were carried out using SPSS version 19.0 (SPSS, Inc., Chicago, IL, USA). The 2-tailed Student’s t-test was used to calculate the difference in the mRNA expression of HOXA1 and cyclin D1 between cancer tissues and paired normal mucosae. The correlation between HOXA1 and cyclin D1 mRNA and protein expression was calculated using Spearman’s correlation coefficient test. The significance of the differences among covariates was determined using two-tailed χ2 or Fisher’s exact tests where appropriate. The Kaplan-Meier method was used to calculate the survival rates, and the log-rank test was used to compare survival curves. Cox proportional hazards models were utilized to investigate the independent risk factors for death, and significant factors were selected for the final multivariate regression model. The in vitro and in vivo data were expressed as mean ± SD and were compared using the 2-tailed Student’s t-test. Differences with P values less than 0.05 were considered statistically significant.

## Results

### HOXA1 is upregulated in GC tissues compared to the levels in corresponding adjacent normal mucosae

**Fig. 1:**
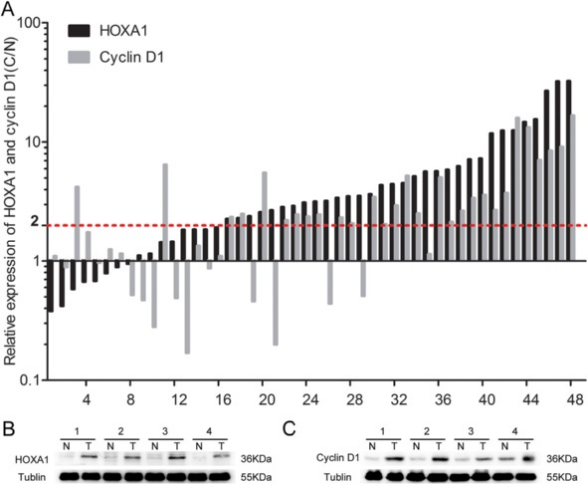
Expression of HOXA1 and cyclin D1 in human gastric cancer (GC) tissues. **a** Relative HOXA1 and cyclin D1 mRNA expression in 48 tumor tissues and paired adjacent normal mucosae as determined by qRT-PCR. An increase (at least 2-fold) in HOXA1 and cyclin D1 mRNA levels compared to noncancerous tissue was observed in 32 (66.7 %) and 29 (60.4 %) of the GC tissue samples. The 2-ΔΔCt method was used to calculate the fold change. **b** Western blotting showed higher HOXA1 protein expression in GC tissues than in the matched adjacent normal mucosae. **c** Western blotting showed that cyclin D1 protein expression was upregulated in GC tissues compared to the levels in the matched adjacent normal mucosae

The mRNA expression of *HOXA1* was examined in 48 GC tissue and paired mucosa samples by qRT-PCR. A majority of the GC samples (32, 66.7 %) showed a ≥2-fold increase in *HOXA1* mRNA levels compared with the levels in adjacent normal mucosae (Fig. 1a). The relative expression (ΔCt) of *HOXA1* mRNA in cancerous tissue was significantly lower than that in normal mucosa (9.57 ± 1.84 vs. 11.23 ± 1.66, respectively; *P* < 0.001). Similarly, western blotting revealed a significant elevation of HOXA1 protein expression in GC tissues compared with the expression in adjacent normal mucosae (Fig. 1b), suggesting that HOXA1 expression was elevated at both the transcriptional and translational levels.

### Knockdown of HOXA1 expression inhibits GC cell proliferation

**Fig. 2:**
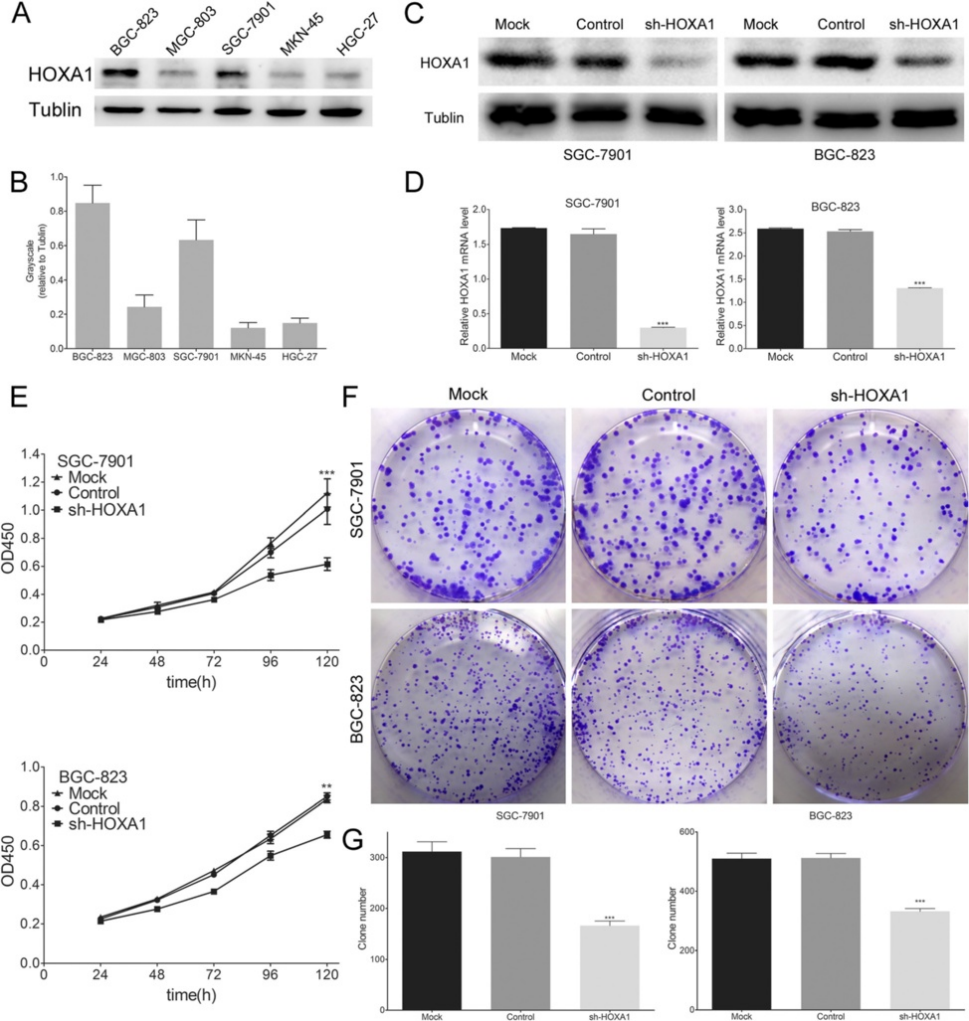
HOXA1 expression in cell lines and the effects of HOXA1 knockdown in GC cell lines. **a** HOXA1 protein levels in five GC cell lines and **b** grayscale values were evaluated. SGC-7901 and BGC-823 showed higher HOXA1 expression than the other tested cell lines. **c** Western blotting analysis of HOXA1 expression in stable HOXA1 knockdown SGC-7901 and BGC-823 cell lines. **d** Real-time PCR analysis of HOXA1 expression in stable HOXA1 knockdown SGC-7901 and BGC-823 cell lines. **e** Effect of HOXA1 knockdown on cell growth was evaluated by the Cell Counting Kit-8 assay. Cell growth was inhibited by HOXA1 knockdown. **f** A plate colony formation assay was performed to assess the impact of HOXA1 knockdown on cell growth. HOXA1 knocked down GC cells showed a weaker clone formation. **g** The number of GC cell clones was quantified. (**P < 0.01, ***P < 0.001)

To explore the function of HOXA1 in GC, we suppressed the expression of HOXA1. Western blotting demonstrated that HOXA1 expression was higher in SGC-7901 and BGC-823 cells than in the other tested cell lines (Fig. 2a, b). The efficiency of shRNA-mediated knockdown in SGC-7901 and BGC-823 cells was confirmed by western blotting and qRT-PCR (Fig. 2c, d). Next, we performed CCK-8 and plate colony formation assays to assess the role of HOXA1 in GC cell growth. As shown in Fig. 2e, significant inhibition of cell growth was observed in the sh-HOXA1 group compared with the growth in the control groups. In addition, the colony formation assay showed that HOXA1 knockdown reduced colony formation compared with that in the control group (*P* < 0.001; Fig. 2f, g). These results indicated that knockdown of HOXA1 expression inhibited GC cell proliferation.

### Knockdown of HOXA1 expression inhibits GC cell migration and invasion

**Fig. 3:**
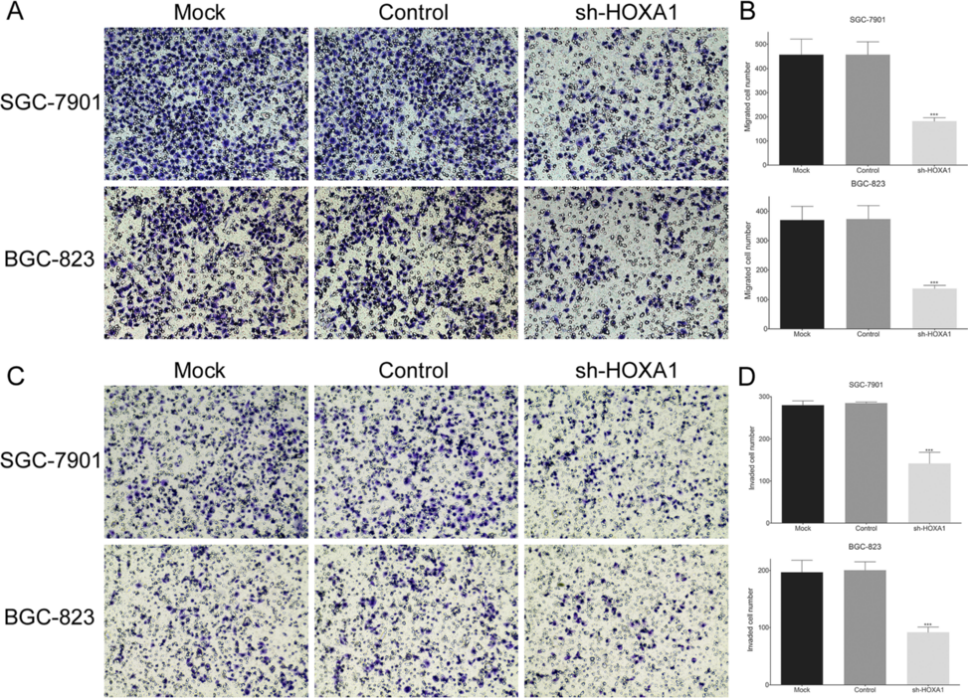
Knockdown of HOXA1 expression inhibits GC cell migration and invasion. **a** The effect of HOXA1 knockdown on the migration of GC cells using transwell chambers without Matrigel. **b** The number of migrated cells was significantly lower in the sh-HOXA1 group than in the control groups (Control and Mock). **c** The effect of HOXA1 knockdown on GC cell invasion using transwell chambers coated with Matrigel. **d** The number of invaded cells was significantly lower in the sh-HOXA1 group than in the control groups (Control and Mock). Original magnification, 200×. (***P < 0.001)

Cell migration and invasion are necessary for tumor development and metastasis. We used transwell chambers either uncoated or precoated with Matrigel to assess the effects of HOXA1 expression on cell migration and invasion, respectively. Knockdown of HOXA1 inhibited the migration and invasion of GC cells compared with that of the control cells (*P* < 0.001; Fig. 3), indicating that HOXA1 increases the migrant and invasive behavior of these cells. These findings indicated that upregulation of HOXA1 may contribute to GC progression by promoting cell migration and invasion.

### Knockdown of HOXA1 expression inhibits xenograft tumor growth

**Fig. 4:**
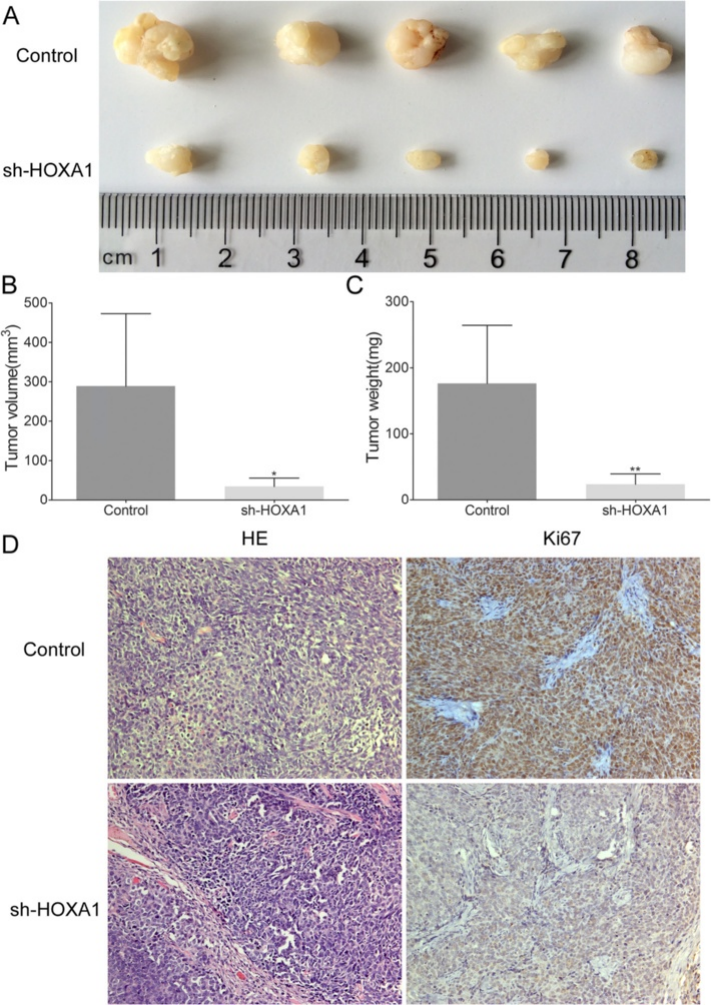
Knockdown of HOXA1 expression suppresses tumorigenicity in vivo. **a** BGC-823-shHOXA1 and BGC-823-Control cells were injected subcutaneously into nude mice. After 3 weeks, the tumors were dissected. The tumors of mice in the BGC-823-shHOXA1 group were smaller than the tumors of BGC-823-Control group. **b** Tumor volume of the BGC-823-Control and BGC-823-shHOXA1 groups 3 weeks after cell injection (*P < 0.05). **c** Tumor weight of the BGC-823-Control and BGC-823-shHOXA1 groups 3 weeks after cell injection (**P < 0.01). **d** H&E- and IHC-stained paraffin-embedded sections obtained from the xenografts. IHC staining showed that the expression of Ki67 was weaker in the BGC-823-shHOXA1 group than in the BGC-823-Control group. Original magnification, 200×

To determine the role of HOXA1 in tumorigenesis, BGC-823-shHOXA1 cells and BGC-823-Control cells were subcutaneously injected into nude mice, and the resulting tumors were isolated 3 weeks later. As shown in Fig. 4, the growth index of the tumors in the BGC-823-shHOXA1 group was significantly lower than that of tumors in the BGC-823-Control group. Moreover, HOXA1 knockdown significantly inhibited overall tumor growth as assessed by the measurements of tumor volume and mass (*P* < 0.05 and *P* < 0.01, respectively; Fig. 4b, c). Particularly, the average tumor volume and weight of the BGC-823-shHOXA1 group were dramatically lower than those of the BGC-823-Control group; tumor volume at 3 weeks was 34.29 ± 21.80 mm^3^ vs. 288.70 ± 183.82 mm^3^, respectively, and tumor weight was 23.80 ± 15.66 mg vs. 176.40 ± 88.25 mg, respectively. In addition, IHC was utilized to detect Ki67, a cellular proliferation marker, in the xenografts. The expression of Ki67 was significantly weaker in the xenografts containing BGC-823-shHOXA1 cells than in xenografts containing BGC-823-Control cells (Fig. 4d). These findings showed that downregulation of HOXA1 expression suppressed GC tumor growth in vivo.

### Knockdown of HOXA1 expression induces changes in the cell cycle of GC cells and decreases cyclin D1 expression

**Fig. 5:**
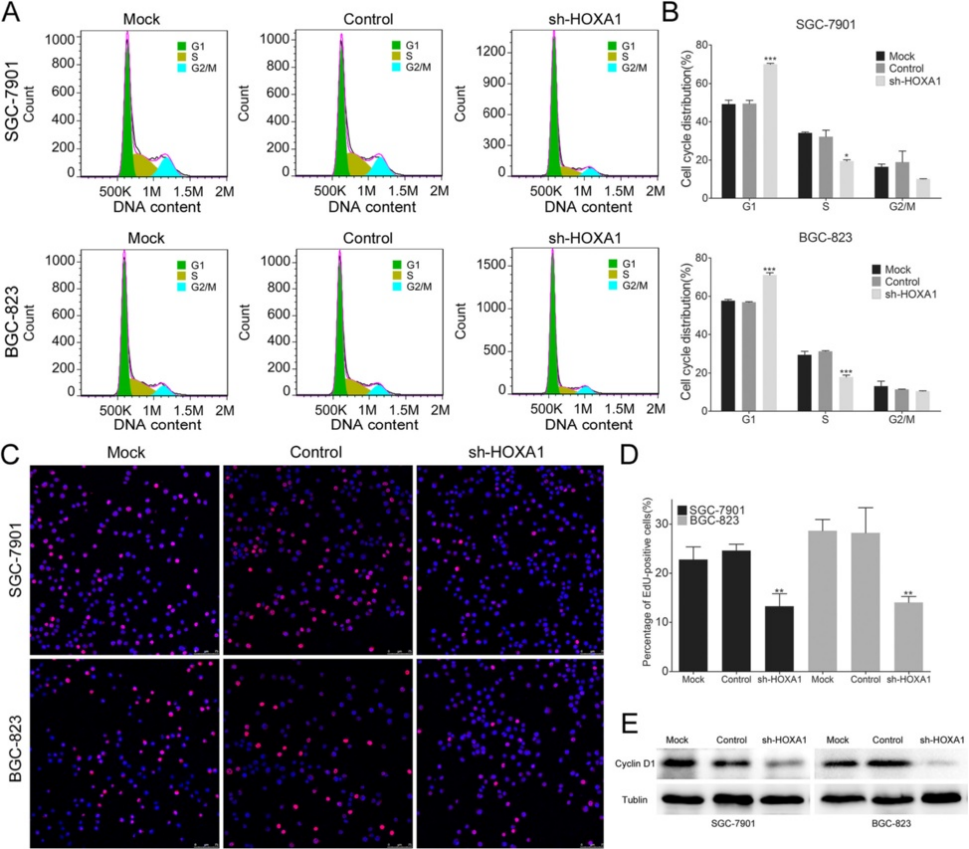
HOXA1 knockdown induces marked cell cycle arrest. **a** Flow cytometry results showing the cell phase distribution of GC cells. HOXA1 knockdown cells (the sh-HOXA1 group) show G1 phase arrest and a reduced number of cells in S phase compared to cells in the control groups. **b** The percentages of cells in each phase were quantified (cell phase distribution). **c** Proliferating SGC-7901 and BGC-823 cells were labeled with EdU and Hoechst33342. EdU staining is shown in red, and Hoechst33342 staining is shown in blue, an overlay of the EdU and Hoechst 33342 stained images are shown. Fewer EdU-positive cells were detected in the sh-HOXA1 group than the control groups, which is indicative of a reduction of the number of cells in S phase in the sh-HOXA1 group compared to that in the control. **d** The percentage of EdU-positive cells was quantified. **e** Western blot analysis of cyclin D1 expression in stable HOXA1 knockdown SGC-7901 and BGC-823 cell lines. (*P < 0.05, **P < 0.01, ***P < 0.001)

Next, we utilized flow cytometry analysis to determine the role of HOXA1 in the cell cycle and found that HOXA1 knockdown cells were arrested in the G1 phase and the number of S phase cells was reduced (Fig. 5a, b). Then, we used an EdU incorporation assay to determine how HOXA1 knockdown affects the number of proliferating cells. The results showed that the number of EdU-positive cells in the sh-HOXA1 group was lower than that in the control group (Fig. 5c, d). As EdU is incorporated into DNA during synthesis in S phase, fewer EdU-positive cells means a reduction of the number of cells in S phase, which is in accord with the cell cycle analysis results. Cyclin D1 has been reported to play an important role in cell cycle progression, mainly via regulation of the G1-S-phase transition [30]. Then, we assessed cyclin D1 expression in our established stable GC cells and found that expression was markedly lower in SGC-7901-shHOXA1 and BGC-823-shHOXA1 cells than in the control cells (Fig. 5e). These results indicated that HOXA1 knockdown induced an accumulation of SGC-7901 and BGC-823 cells in the G1 phase and a reduction in the number of cells in S phase and the expression of cyclin D1.

### Cyclin D1 is upregulated in GC tissues compared with the levels in the corresponding adjacent normal mucosae

We then determined the mRNA expression of *cyclin D1* in GC tissues and paired mucosae. Twenty-nine (60.4 %) of the GC samples showed a ≥2-fold increase in cyclin D1 mRNA levels compared with the levels in the adjacent normal mucosae (Fig. 1a). The relative expression (ΔCt) of *cyclin D1* mRNA in GC was significantly lower than that in normal mucosae (4.74 ± 1.34 vs. 5.77 ± 1.35, respectively; *P* < 0.001). Interestingly, increased *HOXA1* and *cyclin D1* mRNA levels were detected in 27 (56.3 %) GC tissues, and a statistically significant correlation was found (r = 0.651, *P* < 0.001). Similarly, western blotting revealed a significant elevation of cyclin D1 protein expression in GC tissues compared with the levels in the adjacent normal mucosae (Fig. 1c), suggesting that cyclin D1 expression is elevated at both the transcriptional and translational levels.

### Correlation of HOXA1 and cyclin D1 expression with clinicopathological parameters

The expression of HOXA1 and cyclin D1 was assessed by IHC analysis using a TMA of 264 primary GC cases paired with normal mucosae and 104 metastatic lymph nodes.

**Fig. 6:**
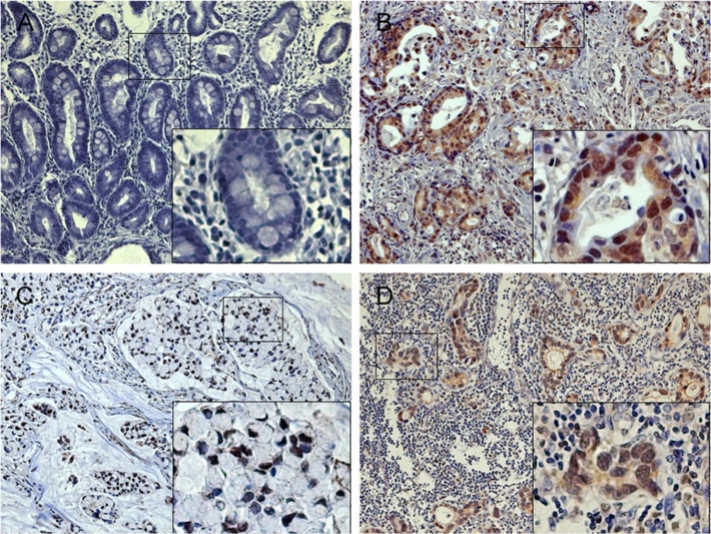
Immunohistochemical staining of HOXA1 expression in normal and gastric cancer tissues. HOXA1 staining was observed mainly in the nuclei of GC cells and in the cytoplasm. HOXA1 expression was much higher in the GC tissues than in the adjacent normal mucosae. Normal gastric tissue showing negative HOXA1 expression (**a**); Positive HOXA1 staining in moderately differentiated GC tissue (**b**), signet ring cell carcinoma (**c**) and metastatic lymph nodes (**d**). Original magnification, 200× (400× for inset images)

We detected significant differences in HOXA1 expression among normal mucosae, GC tissues, and LNM (Table 1, *P* < 0.001). HOXA1 staining was mainly observed in the nuclei of GC cells, and was detected in the cytoplasm (Fig. 6). As shown in Table 1, HOXA1 was obviously upregulated in 54.5 % (144/264) of the primary cancer specimens, whereas HOXA1 was upregulated in only 23.1 % (61/264) of adjacent normal mucosae. HOXA1 expression in LNM (71.2 %) was much higher than that in GC tissues (54.5 %), and this difference was significant (*P* = 0.004). The associations between HOXA1 expression and various clinicopathological parameters are shown in Table 2. Elevated HOXA1 expression was significantly associated with the International Union against Cancer (UICC) stage (*P* < 0.001), invasion depth (*P* = 0.040), nodal involvement (*P* = 0.004), and differentiation (*P* < 0.001; Table 2).

**Table 1:** Expression of HOXA1 and cyclin D1 in normal mucosae, GC tissues, and lymph node metastases

Expression of HOXA1 or cyclin D1	Tissue samples			*P* value
	Normal mucosae (n = 264) (%)	GC tissues (n = 264) (%)	LNM (n = 104) (%)	
HOXA1				
Negative	203 (76.9)	120 (45.5)	30 (28.8)	<0.001^*,**^
Positive	61 (23.1)	144 (54.5)	74 (71.2)	0.004^*,***^
cyclin D1				
Negative	231 (87.5)	127 (48.1)	41 (39.4)	<0.001^*,****^
Positive	33 (12.5)	137 (51.9)	63 (60.6)	0.132
HOXA1/cyclin D1				
Both negative	170 (64.4)	89 (33.7)	24 (23.1)	<0.001^*,*****^
One positive	94 (35.6)	69 (26.1)	23 (22.1)	0.033^*,******^
Both positive	0 (0.00)	106 (40.2)	57 (54.8)	

*GC* gastric cancer

*LNM* lymph node metastases

*P* value calculated using chi-square test or Fisher's exact test

^*^Significant difference

Significant difference in the expression of HOXA1^**^, cyclin D1^****^, or HOXA1/cyclin D1^*****^ among normal mucosae, GC tissues, and LNM

Significant difference in the expression of HOXA1^***^ and HOXA1/cyclin D1^******^ between GC tissues and LNM

**Table 2:** Relationship between clinicopathologic parameters and HOXA1 or cyclin D1 protein expression in gastric cancer (n = 264)

Characteristics	Total	HOXA1 expression		*P* value	Cyclin D1 expression		*P* value
		Negative (120)	Positive (144)		Negative (127)	Positive (137)	
Age(years)				0.457			0.845
<65	121	58	63		59	62	
≥65	143	62	81		68	75	
Gender				0.095			0.712
Male	157	78	79		77	80	
Female	107	42	65		50	57	
T stage				0.040*			0.816
T1	76	44	32		38	38	
T2	42	20	22		22	20	
T3	118	47	71		53	65	
T4	28	9	19		14	14	
N stage				0.004*			0.179
N0	116	67	49		56	60	
N1	91	35	56		48	43	
N2	40	13	27		19	21	
N3	17	5	12		4	13	
M stage				0.355			0.751
M0	254	117	137		123	131	
M1	10	3	7		4	6	
UICC stage				<0.001*			0.400
I	95	54	41		49	46	
II	48	28	20		24	24	
III	89	28	61		43	46	
IV	32	10	22		11	21	
Differentiation				<0.001*			0.044*
High	47	33	14		30	17	
Moderate	42	20	22		21	21	
Low	175	67	108		76	99	

*Significant association among variables (*P* < 0.05)

In addition, significant differences were detected for the cyclin D1 expression in normal mucosae, GC tissues, and LNM (Table 1, *P* < 0.001). Of the 264 primary cancer specimens, 51.9 % (137/264) showed positive cyclin D1 expression, whereas only 12.5 % (33/264) of the adjacent normal mucosae were positive (Table 1). However, no significant difference in cyclin D1 expression was found between GC tissues and LNM (Table 1, *P* = 0.132). Overexpression of cyclin D1 was significantly associated with differentiation (Table 2, *P* = 0.044).

**Fig. 7:**
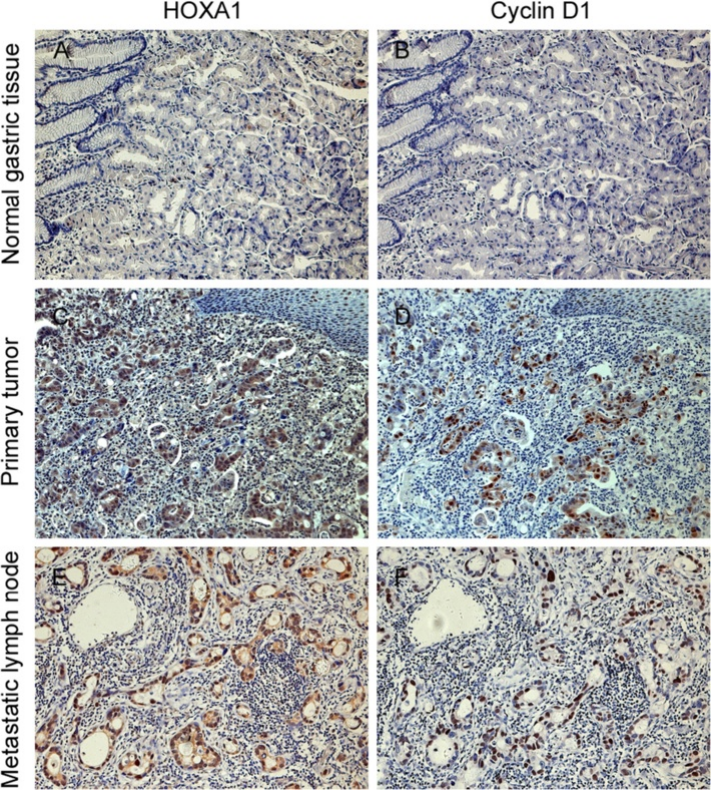
Expression of HOXA1 and cyclin D1 in normal, tumor, and metastatic lymph node tissues. HOXA1 and cyclin D1 expression were detected in the same location in one tumor specimen. Negative HOXA1 and cyclin D1 staining in normal gastric tissue (**a**-**b**). Positive HOXA1 and cyclin D1 staining in GC tissue (**c**-**d**), and metastatic lymph nodes (**e**-**f**). Original magnification, 200×

In addition, of the 144 specimens with positive HOXA1 expression, 73.6 % (106/144) showed positive cyclin D1 expression. We found a statistically significant correlation between HOXA1 and cyclin D1 expression (r = 0.476, *P* < 0.001, Table 3), and HOXA1 and cyclin D1 expression were detected in the same location of one tumor specimen (Fig. 7). These results demonstrated that there was a correlation between HOXA1 and cyclin D1 in GC.

**Table 3:** Association between HOXA1 and cyclin D1 expression in gastric cancer tissues

Sample	Cyclin D1 expression		r	*P* value
	Negative	Positive		
HOXA1 negative	89	31	0.476	<0.001
HOXA1 positive	38	106

### Survival analysis and prognostic significance of HOXA1 and/or cyclin D1 expression

**Fig. 8:**
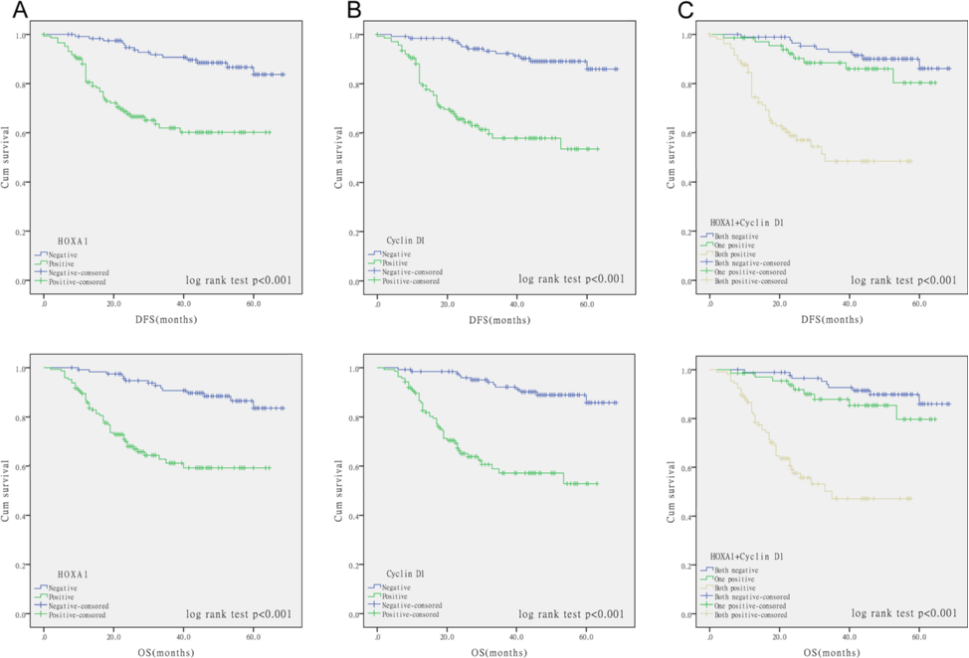
Kaplan-Meier analysis of disease-free survival (DFS) and overall survival (OS) in 264 GC patients. **a** DFS and OS of patients according to HOXA1 expression levels determined by immunohistochemical staining. The survival rate of patients with HOXA1-positive tumors was significantly lower than that of patients with HOXA1-negative tumors (*P* < 0.001). **b** DFS and OS were significantly worse in patients with cyclin D1-positive tumors than in patients with cyclin D1-negative tumors (*P* < 0.001). **c** DFS and OS were significantly lower in patients with HOXA1- and cyclin D1-positive tumors than in patients with HOXA1- and cyclin D1-negative tumors (*P* < 0.001)

To assess the possible association between the expression of HOXA1 and/or cyclin D1 in GC tumors and patient survival, Kaplan-Meier curves and the log-rank test were used to determine DFS and OS in 264 patients who underwent radical gastrectomy (Fig. 8). Patients with HOXA1-positive tumors had a poorer DFS (*P* < 0.001) and OS (*P* < 0.001) than patients with HOXA1-negative tumors (Fig. 8a). Cyclin D1 was also significantly associated with DFS (*P* < 0.001) and OS (*P* < 0.001; Fig. 8b). To assess concomitant HOXA1 and cyclin D1 protein expression, we divided the specimens into three groups: group 1, tumors exhibiting no HOXA1 or cyclin D1 expression (HOXA1-/cyclin D1-, 89 specimens); group 2, tumors with abnormal expression of one protein (HOXA1-/cyclin D1+ or HOXA1+/cyclin D1-, 69 specimens); and group 3, tumors with abnormal expression of both proteins (HOXA1+/cyclin D1+, 106 specimens). Group 1 showed better DFS and OS than group 2, and group 3 showed the worst DFS and OS (*P* < 0.001; Fig. 8c).

In univariate analysis, patients with HOXA1-positive or cyclin D1-positive tumors had a markedly lower OS and DFS than patients with HOXA1-negative or cyclin D1-negative tumors (HOXA1, OS, HR 4.601 [95 % CI 2.504–8.454], *P* < 0.001, DFS, HR 4.438 [95 % CI 2.418–8.145], *P* < 0.001; cyclin D1, OS, HR 5.893 [95 % CI 3.159–10.990], *P* < 0.001, DFS, HR 5.673 [95 % CI 3.044–10.572], *P* < 0.001) (Table 4). Furthermore, lower OS and DFS were also observed in patients with both HOXA1-positive and cyclin D1-positive tumors (OS, HR 9.064 [95 % CI 4.278–19.203], *P* < 0.001; DFS, HR 8.495 [95 % CI 4.021–17.948], *P* < 0.001; Table 4). The multivariate analysis demonstrated that the expression of both HOXA1 and cyclin D1 was an independent prognostic factor for OS (HR 9.905 [95 % CI 4.453–22.031]; *P* < 0.001) and DFS (HR 8.636 95 % CI [3.916–19.047]; *P* < 0.001), but not the expression of HOXA1 or cyclin D1 alone (Table 4).

**Table 4:** Univariate and multivariate analysis for overall survival (OS) and disease-free survival (DFS)

	OS				DFS			
	Univariate		Multivariate		Univariate		Multivariate	
Variable	HR (95 % CI)	*P* value	HR (95 % CI)	*P* value	HR (95 % CI)	*P* value	HR (95 % CI)	*P* value
HOXA1/cyclin D1								
Both negative	1		1		1		1	
One positive	1.623(0.643-4.099)	0.305	1.800(0.702-4.615)	0.221	1.577(0.625-3.983)	0.335	1.674(0.652-4.297)	0.284
Both positive	9.064(4.278-19.203)	<0.001*	9.905(4.453-22.031)	<0.001*	8.495(4.021-17.948)	<0.001*	8.636(3.916-19.047)	<0.001*
HOXA1								
Negative	1				1			
Positive	4.601(2.504-8.454)	<0.001*			4.438(2.418-8.145)	<0.001*		
Cyclin D1								
Negative	1				1			
Positive	5.893(3.159-10.990)	<0.001*			5.673(3.044-10.572)	<0.001*		
Age(years)								
<65	1				1		1	
≥65	1.880(1.103-3.202)	0.020*			1.862(1.093-3.172)	0.022*	1.738(1.011-2.987)	0.046*
Gender								
Male	1				‒			
Female	1.002(0.603-1.666)	0.992			1.006(0.605-1.671)	0.982		
T stage								
T1	1				1			
T2	2.787(0.786-9.885)	0.113			2.871(0.809-10.183)	0.103		
T3	7.596(2.709-21.302)	<0.001*			7.543(2.690-21.150)	<0.001*		
T4	12.061(3.928-37.036)	<0.001*			12.200(3.973-37.465)	<0.001*		
N stage								
N0	1				1			
N1	4.023(1.799-8.999)	0.001*			4.048(1.810-9.054)	0.001*		
N2	9.647(4.238-21.959)	<0.001*			9.489(4.170-21.592)	<0.001*		
N3	16.913(6.668-42.899)	<0.001*			18.152(7.149-46.088)	<0.001*		
M stage								
M0	1				1			
M1	3.082(1.232-7.709)	0.016*			3.405(1.361-8.518)	0.009*		
UICC stage								
I	1		1		1		1	
II	6.795(1.869-24.701)	0.004*	8.168(2.241-29.773)	0.001*	6.867(1.889-24.962)	0.003*	7.172(1.961-26.222)	0.003*
III	13.458(4.100-44.180)	<0.001*	14.327(4.325-47.457)	<0.001*	13.355(4.069-43.834)	<0.001*	12.571(3.791-41.678)	<0.001*
IV	32.066(9.433-109.001)	<0.001*	30.392(8.750-105.567)	<0.001*	34.583(10.172-117.570)	<0.001*	33.649(9.676-117.020)	<0.001*
Differentiation								
High	1				1			
Moderate	5.441(1.155-25.634)	0.032*			5.499(1.167-25.909)	0.031*		
Low	8.506(2.070-34.961)	0.003*			8.581(2.088-35.268)	0.003*		

*HR* hazard ratio; *CI* confidence interval

*P < 0.05 indicates that the 95 % CI of the HR did not include 1

## Discussion

It is well known that tumor cells and embryonic stem cells share some common pathways related to self-renewal and proliferation and that stem cell genes can play direct roles in tumor progression and/or act as valuable markers of tumorigenesis [31, 32]. HOX genes are typical examples of the close relationship between embryogenesis and tumorigenesis [33]. In general, HOX genes are master regulators of embryonic development and stem cell differentiation; however, their misexpression can lead to changes in cancer-associated properties, such as proliferation, migration, invasion, and survival [34, 35]. HOXA1, a HOX gene, is a pivotal transcriptional regulator of early embryonic development that plays a critical role in the development of the brainstem, inner ear, and heart in humans and mice [36, 37]. Overexpression of HOXA1 is associated with a variety of human tumors, including breast, lung, skin, liver, and prostate [18, 19, 21, 38–40]. However, the number of studies on HOXA1 expression during GC tumorigenesis and progression are very limited.

In the present study, we showed for the first time that both HOXA1 mRNA and protein expression were upregulated in primary GC tissues compared with the levels in adjacent normal mucosae. To further elucidate the role of HOXA1 in tumor progression, an shRNA-based strategy was used to stably knockdown HOXA1 expression in SGC-7901 and BGC-823 cells, which typically express high levels of HOXA1 protein. Then, we determined, for the first time, that HOXA1 knockdown inhibited GC cell proliferation using CCK-8 and colony formation assays. Moreover, knockdown of HOXA1 expression also led to a significant reduction in xenograft tumor formation. Recently, Wardwell-Ozgo et al. [18] showed that HOXA1 has a potent effect on cell invasion in melanoma. Meanwhile, Wang et al. [40] reported that HOXA1 knockdown inhibits the growth, invasion, and migration of prostate cancer cells. Coincidentally, our study revealed that HOXA1 knockdown in GC cells suppressed migration and invasion, which indicates the potential involvement of HOXA1 in tumor metastasis. These in vitro and in vivo data suggest that HOXA1 functions in GC cell proliferation, migration, invasion, and tumorigenesis.

Our study showed that knockdown of HOXA1 expression had a marked effect on GC cell proliferation. As the cell cycle is closely related to cell proliferation, we analyzed the effect of HOXA1 knockdown on the cell cycle and found that these cells were arrested in the G1 phase and the number of S phase cells was reduced. Then, we performed an EdU incorporation assay to determine the role of HOXA1 in GC progression. The results of the EdU incorporation assay showed that HOXA1 knockdown suppressed GC cell proliferation. More importantly, as EdU is incorporated into DNA during synthesis, the decreased number of EdU-incorporated SGC-7901-shHOXA1 and BGC-823-shHOXA1 cells suggested that the cell cycle was arrested in the G1 phase, which was validated in our previous cell cycle analysis showing that the number of S phase cells was reduced.

As our data show, downregulation of HOXA1 expression suppressed GC cell proliferation mainly by inhibiting the cell cycle. Recently, HOXA1 was reported to stimulate the transcription of cyclin D1, which increases cell proliferation and survival [21]. Another important experiment indicated that upregulation of HOXA1 activated the transcription of cyclin D1 and promoted the G1-S transition in cancer cells [41]. Cyclin D1 plays an important role in cell cycle progression, mainly by affecting regulation of the G1-S-phase transition [30]. Several studies have identified cyclin D1 as involved early in the development of GC [23, 42, 43]. Then, we assessed the expression of cyclin D1 by western blotting in our established stable GC cells. We found that cyclin D1 expression was dramatically decreased in SGC-7901-shHOXA1 and BGC-823-shHOXA1 cells compared to the levels in the corresponding control cells, which indicates that HOXA1 influences tumor cell proliferation by regulating cyclin D1.

Next, we analyzed the relationship between HOXA1 and cyclin D1 on a histological level. We detected the mRNA and protein expression of cyclin D1 and found that both were higher in primary GC tissues than in normal mucosae. Interestingly, increased HOXA1 and cyclin D1 mRNA levels were detected in 27 (56.3 %) GC tissue samples, and a statistically significant correlation was detected between them (r = 0.651, *P* < 0.001). Then, to evaluate the relationship between HOXA1 expression and clinicopathological parameters, we used IHC on a GC TMA with an expanded number of tissue samples and found that HOXA1 expression was significantly associated with UICC stage, invasion depth, nodal involvement, and differentiation. Furthermore, cyclin D1 was more frequently detected in areas of positive HOXA1 staining in GC tissues, and a significant correlation was found between HOXA1 and cyclin D1 expression (r = 0.476, *P* < 0.001). Thus, the intimate relationship between HOXA1 and cyclin D1 was confirmed at the histological level.

Recent research indicated that HOXA1 expression is markedly correlated with the overall survival of patients with hepatocellular carcinoma [39]. However, the prognostic value of HOXA1 in GC has not been evaluated. According to the work by Wardwell-Ozgo et al., patients with a higher HOXA1 signature had a shorter time to distant metastasis events [18], suggesting the prognostic implications of HOXA1. And our results also showed that HOXA1 could serve as a prognostic marker for GC with a HR of 4.60 for OS and 4.44 for DFS. Intriguingly, survival analysis indicated that HOXA1-and cyclin D1-positive cases had shorter survival time than HOXA1-or cyclin D1-negative cases. What’s more, univariate analysis demonstrated that HOXA1-and cyclin D1-positive patients exhibited a much higher HR for OS (9.06 vs 1.62) and DFS (8.50 vs 1.58) than HOXA1-or cyclin D1-positive patients, indicating that the combination of HOXA1 and cyclin D1 may better prognosticate clinical outcome for GC. Finally, multivariate Cox model analysis confirmed that HOXA1 combined with cyclin D1 was an independent prognostic factor for DFS and OS in GC. Thus, these results suggested that HOXA1, either alone or in combination with cyclin D1, could be a novel prognostic biomarker for GC patients.

## Conclusion

Our research results showed that HOXA1 plays an important role in GC clinical prognosis. First, our data indicated that HOXA1 and cyclin D1 mRNA and protein expression were higher in GC tissues than in the adjacent normal mucosae, and a significant correlation was found between the expression of HOXA1 and cyclin D1. Second, knockdown of HOXA1 expression in GC cells not only inhibited cell proliferation, migration, and invasion and induced changes in the cell cycle in vitro but also suppressed xenograft tumor formation. Moreover, HOXA1 knockdown in GC cells decreased cyclin D1 expression. Third, our clinical data showed that HOXA1, alone or in combination with cyclin D1, may serve as a novel prognostic biomarker for GC. Future studies will focus on the mechanism underlying the role of HOXA1 in the progression of GC and the potential for targeting HOXA1 in GC treatment.

## Abbreviations

GC: gastric cancer
TMA: tissue microarrays
DFS: disease-free survival
OS: overall survival
UICC: the International Union against Cancer
FBS: fetal bovine serum
qRT-PCR: quantitative real-time PCR
SDS-PAGE: sodium dodecyl sulfate-polyacrylamide gel electrophoresis
CCK-8 assay: Cell Counting Kit-8 assay
EdU incorporation assay: 5-Ethynyl-2'- deoxyuridine incorporation assay
IHC: immunohistochemistry
IS: immunostaining score
